# Efficient Power Characteristic Analysis and Multi-Objective Optimization for an Irreversible Simple Closed Gas Turbine Cycle

**DOI:** 10.3390/e24111531

**Published:** 2022-10-26

**Authors:** Xingfu Qiu, Lingen Chen, Yanlin Ge, Shuangshuang Shi

**Affiliations:** 1 Institute of Thermal Science and Power Engineering, Wuhan Institute of Technology, Wuhan 430205, China; 2 Hubei Provincial Engineering Technology Research Center of Green Chemical Equipment, Wuhan 430205, China; 3 School of Mechanical & Electrical Engineering, Wuhan Institute of Technology, Wuhan 430205, China

**Keywords:** irreversible closed gas turbine cycle, finite-time thermodynamics, pressure ratio, heat conductance distribution, NSGA-II algorithm, multi-objective optimization

## Abstract

On the basis of the established irreversible simple closed gas turbine cycle model, this paper optimizes cycle performance further by applying the theory of finite-time thermodynamics. Dimensionless efficient power expression of the cycle is derived. Effects of internal irreversibility (turbine and compressor efficiencies) and heat reservoir temperature ratio on dimensionless efficient power are analyzed. When total heat conductance of two heat exchangers is constant, the double maximum dimensionless efficient power of a cycle can be obtained by optimizing heat-conductance distribution and cycle pressure-ratio. Through the NSGA-II algorithm, multi-objective optimizations are performed on the irreversible closed gas turbine cycle by taking five performance indicators, dimensionless power density, dimensionless ecological function, thermal efficiency, dimensionless efficient power and dimensionless power output, as objective functions, and taking pressure ratio and heat conductance distribution as optimization variables. The Pareto frontiers with the optimal solution set are obtained. The results reflect that heat reservoir temperature ratio and compressor efficiency have greatest influences on dimensionless efficient power, and the deviation indexes obtained by TOPSIS, LINMAP and Shannon Entropy decision-making methods are 0.2921, 0.2921, 0.2284, respectively, for five-objective optimization. The deviation index obtained by Shannon Entropy decision-making method is smaller than other decision-making methods and its result is more ideal.

## 1. Introduction

Since the inception of finite-time thermodynamics (FTT) theory, some scholars have conducted in-depth research in this new branch and made great progress [1,2,3,4,5]. Some have introduced FTT theory to search optimal configurations of cycles and devices, including commercial engine [6], internal combustion engines [7,8], heat exchange system [9], hydraulic recuperation system [10], Stirling engines [11,12], refrigerators [13,14,15], chemical reactor [16], finite source heat engines [17,18], etc. Others have introduced FTT theory to search optimal performances of cycles and devices, including Atkinson cycles [19,20], Otto cycles [21,22], Brayton cycles [23,24], Carnot cycles [25,26,27], Dual cycles [28,29], Stirling cycles [30,31], thermal Brownian cycles [32,33,34], porous medium cycle [35], combined cycles [36,37], etc. Analyzing and optimizing heat engine (HEG) cycle performances with different objectives is an important research work in the FTT field. Salamon and Nitzan [38] investigated profit rate, exergy loss and exergy efficiency of endo-reversible Carnot HEG, besides thermal efficiency (η) and power output (P) optimization objectives (OOs). Sahin et al. [39] provided power density ($P_{d}$) OO for a reversible closed gas turbine (CGT) cycle. Angulo-Brown [40] put forward an ecological function (E) OO for endo-reversible Carnot HEG. Yan [41] used a product of P and η as OO to perform optimization on the endo-reversible Carnot heat engine cycle. Yilmaz [42] named this OO as efficient power ($E_{P}$) and pointed out that HEG designed at the maximum $E_{P}$ condition has better P than that at the maximum $P_{d}$ condition.

In the FTT studies of simple CGT cycles, Refs. [43,44] studied P and η performances of endoreversible CGT cycles with constant- [43] and variable- [44] temperature heat reservoirs; Refs. [45,46,47,48] studied P and η performances of irreversible CGT cycles; Refs. [49,50] studied E performances of endo-reversible [49] and irreversible [50] CGT cycles; Refs. [51,52] studied $P_{d}$ performances of endo=reversible [51] and irreversible [52] CGT cycles. Besides, Arora et al. [53] investigated $E_{P}$ of open cycle Brayton HEG with variable specific heat of working fluid and compared the results with those obtained under maximum P and maximum $P_{d}$ conditions. Because the pressure losses have great influence on open gas turbine cycles [54,55,56,57] and little influence on CGT cycles, they are not considered in the CGT cycle models.

With the increase in OOs, there may be conflicts among different OOs. In order to coordinate the conflicts among OOs, some scholars used NSGA-II [58,59,60,61,62,63,64,65,66,67,68] to perform multi-objective optimization (MOO) for various HEG cycles. Ahmadi et al. [58] studied the applicability of the Stirling-Otto combined cycle and performed MOO on P and η for combined cycle with six decision variables. Zang et al. [59,60] studied $P_{d}$ of porous media cycles with constant specific-heat [59] and linear variable-specific-heat [60], respectively, and carried out MOO on E, η, P and $P_{d}$. Xu et al. [61] performed MOO on E, η, P and $E_{P}$ for Stirling HEG cycle with heat transfer loss and mechanical losses. Wu et al. [62] performed MOO on E, P, $E_{P}$ and η for a magnetohydrodynamic cycle. He et al. [63] performed MOO on E, P, $E_{P}$ and η for electronic HEG considering heat leakage loss, while Qiu et al. [64] studied $E_{P}$ performance of endo-reversible CGT, and performed MOO with five OOs of E, η, P, $P_{d}$ and $E_{P}$.

As of now, there is no open literature concerning $E_{P}$ analysis and MOO for simple irreversible CGT cycle. Taking the maximum $E_{P}$ [41,42,53,64] as OO, although P of HEGs is sacrificed, η of the HEGs is greatly improved and the $E_{P}$ reflects compromise between P and η of the HEGs. MOO [58,59,60,61,62,63,64,65,66,67,68] can weigh the conflicts among different OOs and the MOO algorithm can be used to find the optimal solution when multiple OOs coexist, so as to optimize performance of the HEGs. Based on the simple irreversible CGT cycle model established in Refs. [46,52], this paper will take cycle dimensionless $E_{P}$ (${\bar{E}}_{P}$) as OO and analyze impacts of compressor internal efficiency, turbine internal efficiency, cycle heat reservoir temperature ratio and total heat conductance (HTC) of two heat exchangers (HEXs) on ${\bar{E}}_{P}$ performance. The dimensionless E ($\bar{E}$), η, dimensionless P ($\bar{P}$), ${\bar{E}}_{P}$ and dimensionless $P_{d}$ (${\bar{P}}_{d}$) will be introduced, cycle pressure ratio (π) and distribution (u) of hot-side HEX HTC will be taken as optimization variables to optimize the cycle performance under different combinations of single-objective, two-objectives, three-objectives, four-objectives and five-objectives, and Pareto frontiers will be gained by using NSGA-II. TOPSIS, Shannon Entropy and LINMAP decision-making methods will be adopted to compare deviation indexes under different OO combinations and the best design scheme will be gained.

## 2. Cycle Model

**Figure 1:**
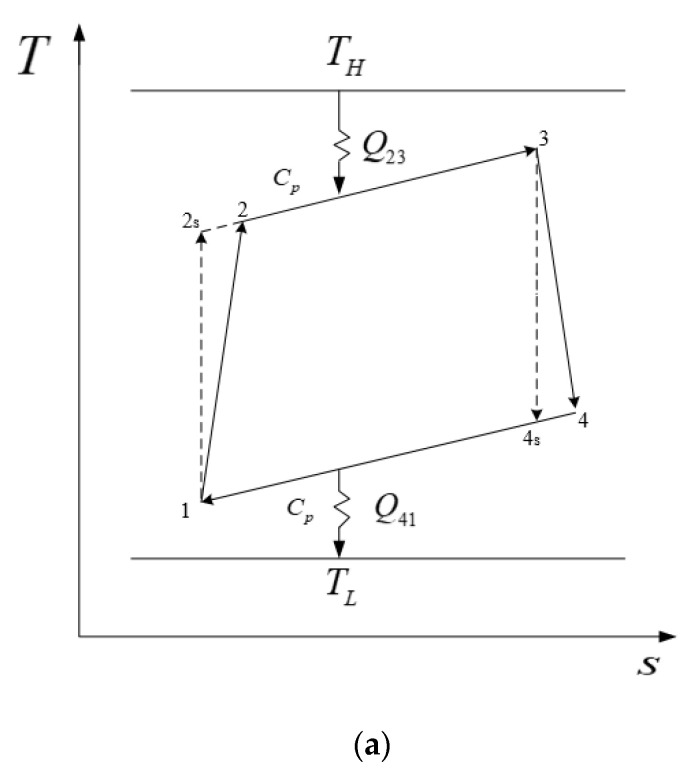

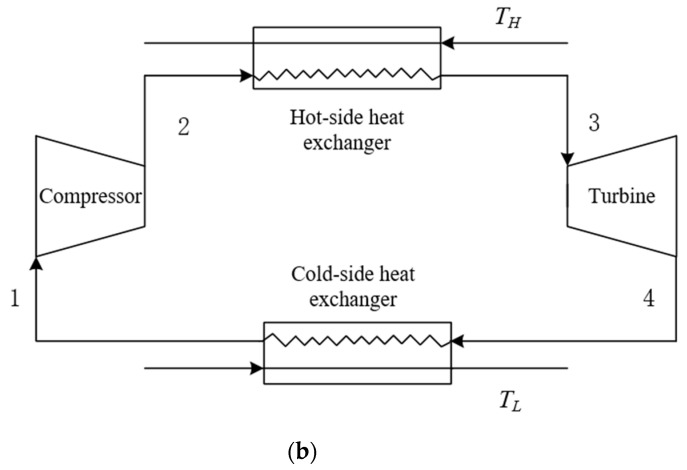
Cycle model. (**a**) *T*-*s* diagram for irreversible simple closed gas turbine cycle [46,52]. (**b**) system diagram for irreversible simple closed gas turbine cycle [64].

Figure 1a gives T−s diagram of an irreversible simple CGT cycle with constant-temperature heat reservoirs [46,52]. $Q_{41}$ (or $Q_{23}$) is heat release (or absorption) rate, $T_{L}$ (or $T_{H}$) is heat sink (or source) temperature. $1\to 2_{s}\to 3\to 4_{s}\to 1$ is the endoreversible cycle, while 1→2→3→4→1 is the actual irreversible one. Figure 1b gives a system diagram of an irreversible simple CGT cycle [64]. The working fluid changes from states 2 to 3 through hot-side HEX, from states 3 to 4 through irreversible expansion of turbine, from states 4 to 1 through cold-side HEX, and finally from states 1 to 2 through irreversible compression of the compressor to complete the whole cycle.

Assuming that thermal capacity rate ($C_{wf}$) of the working medium is constant; $U_{L}$(or $U_{H}$) is HTC of cold- (or hot-) side HEX, and $U_{T}$($U_{T}=U_{H}+U_{L}$) represents the total HTC. Defining HTC distribution (u) as $u=U_{H}/U_{T}$, then there are

(1) $$U_{H}=uU_{T},U_{L}=(1-u)U_{T}$$

The irreversible cycle takes into account losses of compressor and turbine, expressed by the internal efficiencies $\eta_{c}$ and $\eta_{t}$, and there are

(2) $$\eta_{c}=(T_{2s}-T_{1})/(T_{2}-T_{1})$$

(3) $$\eta_{t}=(T_{3}-T_{4})/(T_{3}-T_{4s})$$

From Refs. [46,52], it can be seen that $Q_{23}$ and $Q_{41}$ are

(4) $$Q_{23}=C_{wf}(T_{3}-T_{2})=C_{wf}E_{H}(T_{H}-T_{2})$$

(5) $$Q_{41}=C_{wf}(T_{4}-T_{1})=C_{wf}E_{L}(T_{4}-T_{L})$$

where $E_{L}$ and $E_{H}$ are cold-side and hot-side HEXs effectiveness and these are

(6) $$E_{H}=1-\exp(-N_{H}),E_{L}=1-\exp(-N_{L})$$

where $N_{L}$ and $N_{H}$ are numbers of heat transfer units of cold-side and hot-side HEXs, and these are

(7) $$N_{H}=U_{H}/C_{wf},N_{L}=U_{L}/C_{wf}$$

According to the second law of thermodynamics, for the endoreversible part of the cycle, $1\to 2_{s}\to 3\to 4_{s}\to 1$, the entropy change is zero, and one has

(8) $$c_{p}\ln\frac{T_{3}}{T_{2s}}-c_{p}\ln\frac{T_{4s}}{T_{1}}=0$$

Simplifying Equation (8), there is

(9) $$T_{1}T_{3}=T_{2s}T_{4s}$$

Defining cycle pressure ratio as π, then one has

(10) $$\pi^{m}={\left(\frac{p_{2}}{p_{1}}\right)}^{m}=\frac{T_{2s}}{T_{1}}=\frac{T_{3}}{T_{4s}}$$

where m=(k−1)/k and k is specific heat ratio.

The entropy production rate of irreversible CGT cycle is

(11) $$S_{g}=\frac{Q_{41}}{T_{L}}-\frac{Q_{23}}{T_{H}}$$

For constant pressure process 4→1 of the cycle, there is

(12) $$\frac{v_{4}}{v_{1}}=\frac{T_{4}}{T_{1}}=\frac{T_{4}}{T_{2s}}\cdot \frac{T_{2s}}{T_{1}}=\pi^{m}\frac{T_{4}}{T_{2s}}$$

where $v_{4}$ is the maximum specific volume in the irreversible simple CGT cycle.

From Equations (2)–(5) and (10)–(12), P and η expressions of irreversible CGT cycle are

(13) $$P=Q_{23}-Q_{41}=\frac{\begin{array}{c} C_{wf}\{\{\eta_{c}-(1+\eta_{t}\pi^{-m}-\eta_{t})[(\pi^{m}+\eta_{c}-1)(1-E_{L})+\eta_{c}E_{L}]\}E_{H}T_{H}-\\ \left\{(\pi^{m}+\eta_{c}-1)[(1+\eta_{t}\pi^{-m}-\eta_{t})(1-E_{H})+E_{H}]-\eta_{c}\}E_{L}T_{L}\right\} \end{array}}{\eta_{c}-(\pi^{m}+\eta_{c}-1)(1-E_{H})(1-E_{L})(1+\eta_{t}\pi^{-m}-\eta_{t})}$$

(14) $$\eta =1-Q_{23}/Q_{41}=1-\frac{\begin{array}{c} E_{L}\{(1+\eta_{t}\pi^{-m}-\eta_{t})\eta_{c}E_{H}T_{H}-[\eta_{c}+(\pi^{m}-1\\ +\eta_{c})(1-E_{H})(1+\eta_{t}\pi^{-m}-\eta_{t})]T_{L}\} \end{array}}{\begin{array}{c} E_{H}\{[\eta_{c}-(\pi^{m}+\eta_{c}-1)(1-E_{L})(1+\\ \eta_{t}\pi^{-m}-\eta_{t})]T_{H}-(\pi^{m}+\eta_{c}-1)E_{L}T_{L}\} \end{array}}$$

$P_{d}$ and E expressions of irreversible CGT cycle can be obtained as

(15) $$E=P-T_{0}S_{g}=\frac{\begin{array}{cc} \; & C_{wf}\{\{\left\{\eta_{c}-\left(1+\eta_{t}\pi^{-m}-\eta_{t}\right)\left[\left(\pi^{m}+\eta_{c}-1\right)\left(1-E_{L}\right)+\eta_{c}E_{L}\right]\right\}T_{H}+[\eta_{c}-(1-\\ \; & \eta_{t}+\eta_{t}\pi^{-m})\left(\pi^{m}+\eta_{c}-1\right)]T_{0}\}E_{H}-\{\{\left(\pi^{m}+\eta_{c}-1\right)[\left(1+\eta_{t}\pi^{-m}-\eta_{t}\right)(1-\\ \; & E_{H})+E_{H}]-\eta_{c}\}T_{L}-\left[\eta_{c}-\left(1+\eta_{t}\pi^{-m}-\eta_{t}\right)\left(\pi^{m}+\eta_{c}-1\right)\right]T_{0}\}E_{L}-\\ \; & \left\{\left[\eta_{c}\tau -2\left(\pi^{m}+\eta_{c}-1\right)\right]\left(1+\eta_{t}\pi^{-m}-\eta_{t}\right)+\tau^{-1}\left(\pi^{m}+\eta_{c}-1\right)\right\}E_{H}E_{L}T_{0}\} \end{array}}{\eta_{c}-\left(\pi^{m}+\eta_{c}-1\right)\left(1-E_{H}\right)\left(1-E_{L}\right)\left(1+\eta_{t}\pi^{-m}-\eta_{t}\right)}$$

(16) $$P_{d}=(Q_{23}-Q_{41})/v_{4}=\frac{\begin{array}{c} C_{wf}\{\{\eta_{c}-(1+\eta_{t}\pi^{-m}-\eta_{t})[(\pi^{m}+\eta_{c}-1)(1-E_{L})+\eta_{c}E_{L}]\}E_{H}T_{H}-\\ \left\{(\pi^{m}+\eta_{c}-1)[(1+\eta_{t}\pi^{-m}-\eta_{t})(1-E_{H})+E_{H}]-\eta_{c}\}E_{L}T_{L}\right\} \end{array}}{[\eta_{c}-(\pi^{m}+\eta_{c}-1)(1-E_{H})(1-E_{L})(1+\eta_{t}\pi^{-m}-\eta_{t})]v_{4}}$$

where $T_{0}$ is the ambient temperature.

According to the $E_{P}$ defined in Refs. [41,42], there is

(17) $$E_{P}=P\eta =\frac{\begin{array}{c} C_{wf}\{E_{H}E_{L}\left\{\left(1+\eta_{t}\pi^{-m}-\eta_{t}\right)\left[\left(\pi^{m}+\eta_{c}-1\right)\left(T_{H}+1\right)-T_{H}\eta_{c}\right]-T_{L}\left(\pi^{m}+\eta_{c}-1\right)\right\}+[\eta_{c}-\\ \left(1+\eta_{t}\pi^{-m}-\eta_{t}\right)\left(\pi^{m}+\eta_{c}-1\right)]\left(T_{H}E_{H}+E_{L}\right)\}\{\{\eta_{c}-\left(1+\eta_{t}\pi^{-m}-\eta_{t}\right)[\left(\pi^{m}+\eta_{c}-1\right)\\ \left(1-E_{L}\right)+\eta_{c}E_{L}]\}E_{H}T_{H}-T_{L}\left\{\left(\pi^{m}+\eta_{c}-1\right)\left[\left(1+\eta_{t}\pi^{-m}-\eta_{t}\right)\left(1-E_{H}\right)+E_{H}\right]-\eta_{c}\right\}E_{L}\} \end{array}}{\begin{array}{c} E_{H}\left[\eta_{c}-\left(\pi^{m}+\eta_{c}-1\right)\left(1-E_{H}\right)\left(1-E_{L}\right)\left(1+\eta_{t}\pi^{-m}-\eta_{t}\right)\right]\{[\eta_{c}-\\ \left(\pi^{m}+\eta_{c}-1\right)\left(1-E_{L}\right)\left(1+\eta_{t}\pi^{-m}-\eta_{t}\right)]T_{H}-T_{L}\left(\pi^{m}+\eta_{c}-1\right)E_{L}\} \end{array}}$$

Defining dimensionless P, $P_{d}$, E and $E_{P}$ as: $\bar{P}=P/C_{wf}T_{L}$, ${\bar{P}}_{d}=P_{d}/(C_{wf}T_{L}/v_{1})$, $\bar{E}=E/C_{wf}T_{L}$, and ${\bar{E}}_{P}={\bar{E}}_{P}/C_{wf}T_{L}$, respectively, then there are

(18) $$\bar{P}=\frac{\begin{array}{c} \{\{\eta_{c}-(1+\eta_{t}\pi^{-m}-\eta_{t})[(\pi^{m}+\eta_{c}-1)(1-E_{L})+\eta_{c}E_{L}]\}E_{H}\tau \\ -\{(\pi^{m}+\eta_{c}-1)[(1+\eta_{t}\pi^{-m}-\eta_{t})(1-E_{H})+E_{H}]-\eta_{c}\}E_{L}\} \end{array}}{\eta_{c}-(\pi^{m}+\eta_{c}-1)(1-E_{H})(1-E_{L})(1+\eta_{t}\pi^{-m}-\eta_{t})}$$

(19) $$\bar{E}=\frac{\begin{array}{cc} \; & \{\left\{\eta_{c}-\left(1_{t}+\eta_{t}\pi^{-m}-\eta \right)\left[\left(\pi^{m}+\eta_{c}-1\right)\left(1-E_{L}\right)+\eta_{c}E_{L}\right]\right\}\tau +[\eta_{c}-(1-\eta_{t}\\ \; & +\eta_{t}\pi^{-m})\left(\pi^{m}+\eta_{c}-1\right)]T_{0}/T_{L}\}E_{H}-\{\{\left(\pi^{m}+\eta_{c}-1\right)[\left(1+\eta_{t}\pi^{-m}-\eta_{t}\right)(1-\\ \; & E_{H})+E_{H}]-\eta_{c}\}-\left[\eta_{c}-\left(1+\eta_{t}\pi^{-m}-\eta_{t}\right)\left(\pi^{m}+\eta_{c}-1\right)\right]T_{0}/T_{L}\}E_{L}-\\ \; & \left\{\left[\eta_{c}\tau -2\left(\pi^{m}+\eta_{c}-1\right)\right]\left(1+\eta_{t}\pi^{-m}-\eta_{t}\right)+\tau^{-1}\left(\pi^{m}+\eta_{c}-1\right)\right\}E_{H}E_{L}T_{0}/T_{L} \end{array}}{\eta_{c}-\left(\pi^{m}+\eta_{c}-1\right)\left(1-E_{H}\right)\left(1-E_{L}\right)\left(1+\eta_{t}\pi^{-m}-\eta_{t}\right)}$$

(20) $${\bar{P}}_{d}=\frac{\begin{array}{l} \{\{\eta_{c}-(1+\eta_{t}\pi^{-m}-\eta_{t})[(\pi^{m}+\eta_{c}-1)(1-E_{L})+\eta_{c}E_{L}]\}E_{H}\tau -\{(\pi^{m}+\eta_{c}-1)[(1\\ +\eta_{t}\pi^{-m}-\eta_{t})(1-E_{H})+E_{H}]-\eta_{c}\}E_{L}\}[(1-E_{L})(1+\eta_{t}\pi^{-m}-\eta_{t})E_{H}\tau +E_{L}]\eta_{c} \end{array}}{\begin{array}{l} {}[\eta_{c}-(\pi^{m}+\eta_{c}-1)(1-E_{H})(1-E_{L})(1+\eta_{t}\pi^{-m}-\eta_{t})]\{(1+\\ \eta_{t}\pi^{-m}-\eta_{t})[\eta_{c}E_{H}\tau +(1-E_{H})(\pi^{m}+\eta_{c}-1)E_{L}]\} \end{array}}$$

(21) $${\bar{E}}_{P}=\frac{\begin{array}{c} \{E_{H}E_{L}\left\{\left(1+\eta_{t}\pi^{-m}-\eta_{t}\right)\left[\left(\pi^{m}+\eta_{c}-1\right)\left(\tau +1\right)-\tau \eta_{c}\right]-\left(\pi^{m}+\eta_{c}-1\right)\right\}+[\eta_{c}-(1-\eta_{t}\\ +\eta_{t}\pi^{-m})\left(\pi^{m}+\eta_{c}-1\right)]\left(\tau E_{H}+E_{L}\right)\}\{\{\eta_{c}-\left(1+\eta_{t}\pi^{-m}-\eta_{t}\right)[\left(\pi^{m}+\eta_{c}-1\right)\\ \left(1-E_{L}\right)+\eta_{c}E_{L}]\}E_{H}\tau -\left\{\left(\pi^{m}+\eta_{c}-1\right)\left[\left(1+\eta_{t}\pi^{-m}-\eta_{t}\right)\left(1-E_{H}\right)+E_{H}\right]-\eta_{c}\right\}E_{L}\} \end{array}}{\begin{array}{cc} \; & E_{H}\left[\eta_{c}-\left(\pi^{m}+\eta_{c}-1\right)\left(1-E_{H}\right)\left(1-E_{L}\right)\left(1+\eta_{t}\pi^{-m}-\eta_{t}\right)\right]\{[\eta_{c}-\\ \; & \left(\pi^{m}+\eta_{c}-1\right)\left(1-E_{L}\right)\left(1+\eta_{t}\pi^{-m}-\eta_{t}\right)]\tau -\left(\pi^{m}+\eta_{c}-1\right)E_{L}\} \end{array}}$$

where $\tau =T_{H}/T_{L}$ is the heat reservoir temperature ratio of the irreversible CGT cycle.

When there is no loss of compressor and turbine, that is, when $\eta_{c}=\eta_{t}=1$, Equations (18)–(21) can be changed into

(22) $$\bar{P}=\frac{E_{H}E_{L}[\tau (1-\pi^{-m})+(1-\pi^{m})]}{E_{L}+E_{H}-E_{L}E_{H}}$$

(23) $${\bar{P}}_{d}=\frac{E_{H}E_{L}[(1-\pi^{-m})\tau -\pi^{m}+1][(1-E_{L})E_{H}\tau +E_{L}\pi^{m}]}{\left(E_{L}+E_{H}-E_{L}E_{H})[(1-E_{H})E_{L}\pi^{m}+E_{H}\tau \right]}$$

(24) $$\bar{E}=\frac{E_{H}E_{L}[\tau (1-\pi^{-m})+(1-\pi^{m})-(\tau \pi^{-m}-\tau^{-1}\pi^{m}+2)T_{0}/T_{L}]}{E_{L}+E_{H}-E_{L}E_{H}}$$

(25) $${\bar{E}}_{P}=\frac{E_{H}E_{L}(1-\pi^{-m})[\tau (1-\pi^{-m})+(1-\pi^{m})]}{E_{L}+E_{H}-E_{L}E_{H}}$$

Those are the results of the endo-reversible CGT cycle [43,49,51,52].

## 3. Efficient Power Performance Analyses

In the calculations of this paper, $T_{0}=300\;K$, $T_{L}=310\;K$, $\eta_{c}=\eta_{t}=0.9$, k=1.4, $C_{wf}=1\;\mathrm{kW}/K$ and $U_{T}=10\;\mathrm{kW}/K$ are set.

**Figure 2:**
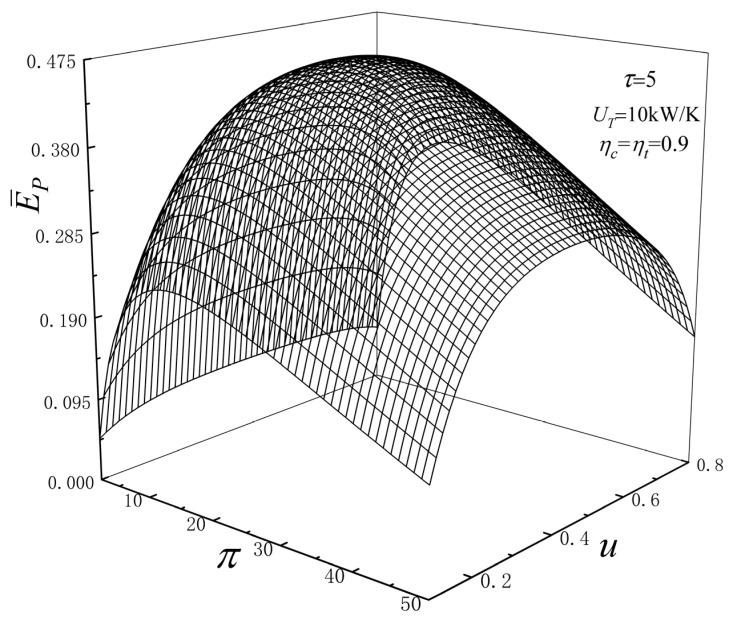
Relations of ${\bar{E}}_{P}$ versus u and π.

Figure 2 reflects the relationships of ${\bar{E}}_{P}$ versus u and π when $\eta_{c}=\eta_{t}=0.9$, $U_{T}=10\;\mathrm{kW}/K$, τ=5. From Figure 2, when u is constant, the ${\bar{E}}_{P}$ and π are parabolic-likes, and there is an optimal u ($u_{opt}$) to achieve maximum the ${\bar{E}}_{P}$ (${\bar{E}}_{P_{\max}}$). When π is constant, the ${\bar{E}}_{P}$ and u are parabolic-likes, and there is an optimal π ($\pi_{opt}$) to achieve ${\bar{E}}_{P_{\max}}$. Therefore, there is a pair of optimal variables ($u_{opt}$ and $\pi_{opt}$), which make the cycle dimensionless $E_{P}$ reach double maximum. At this time, $u_{opt}=0.4716$, $\pi_{opt}=17.4295$, and the double maximum dimensionless E (${\bar{E}}_{P_{\max,2}}$) is 0.4615.

**Figure 3:**
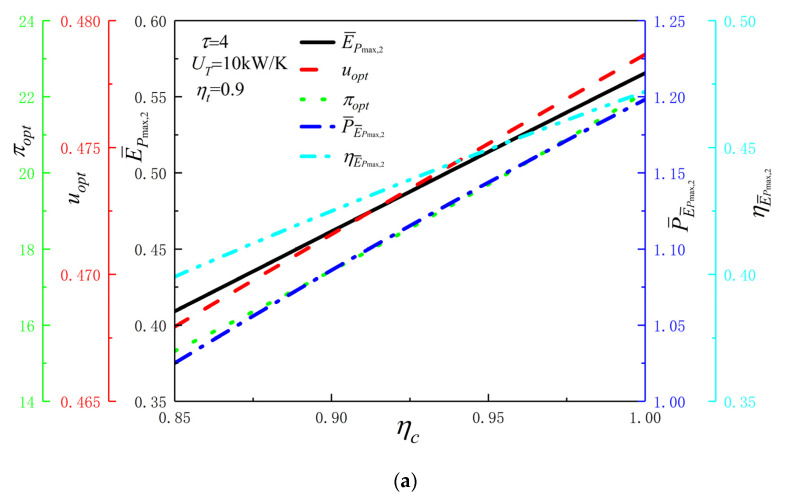

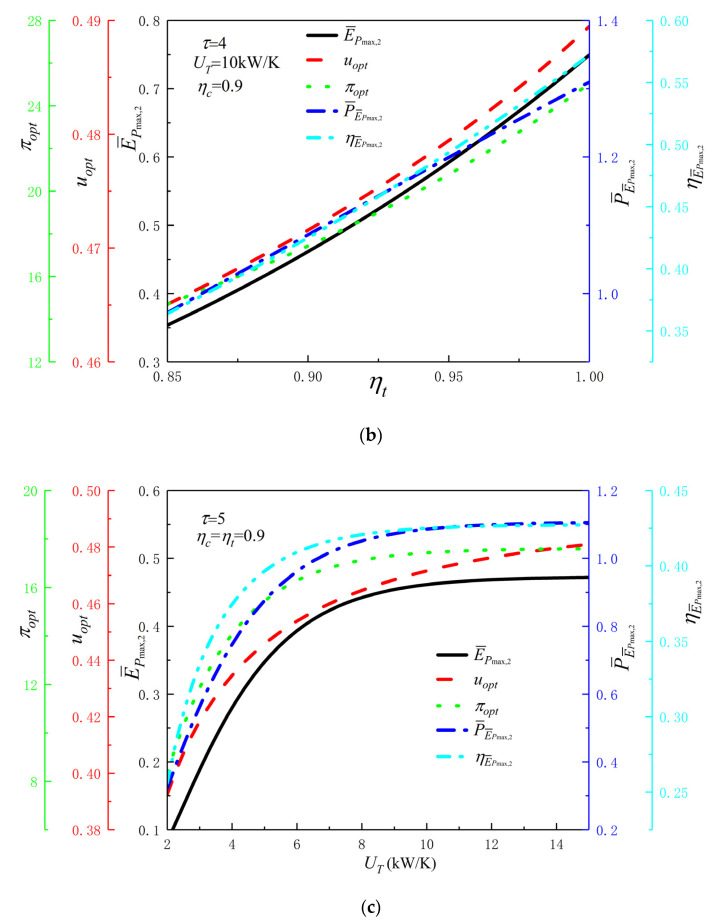

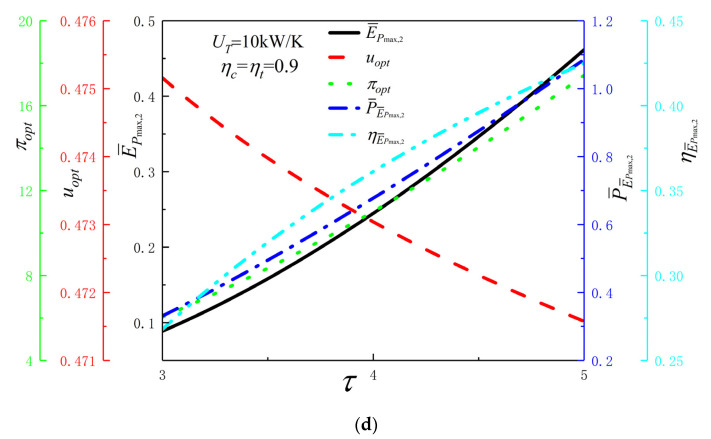
Corresponding $u_{opt}$, $\pi_{opt}$, ${\bar{P}}_{{\bar{E}}_{P_{\max,2}}}$ and $\eta_{{\bar{E}}_{P_{\max,2}}}$ vs. variables. (**a**) ${\bar{E}}_{P_{\max,2}}$, corresponding $u_{opt}$, $\pi_{opt}$, ${\bar{P}}_{{\bar{E}}_{P_{\max,2}}}$ and $\eta_{{\bar{E}}_{P_{\max,2}}}$ vs. $\eta_{c}$; (**b**) ${\bar{E}}_{P_{\max,2}}$, corresponding $u_{opt}$, $\pi_{opt}$, ${\bar{P}}_{{\bar{E}}_{P_{\max,2}}}$ and $\eta_{{\bar{E}}_{P_{\max,2}}}$ vs. $\eta_{t}$; (**c**) ${\bar{E}}_{P_{\max,2}}$, corresponding $u_{opt}$, $\pi_{opt}$, ${\bar{P}}_{{\bar{E}}_{P_{\max,2}}}$ and $\eta_{{\bar{E}}_{P_{\max,2}}}$ vs. $U_{T}$; (**d**) ${\bar{E}}_{P_{\max,2}}$, corresponding $u_{opt}$, $\pi_{opt}$, ${\bar{P}}_{{\bar{E}}_{P_{\max,2}}}$ and $\eta_{{\bar{E}}_{P_{\max,2}}}$ vs. τ.

Figure 3a reflects the ${\bar{E}}_{P_{\max,2}}$, the corresponding $u_{opt}$, $\pi_{opt}$, dimensionless P(${\bar{P}}_{{\bar{E}}_{P_{\max,2}}}$) and η($\eta_{{\bar{E}}_{P_{\max,2}}}$) vs. $\eta_{c}$ when $\eta_{t}=0.9$, $U_{T}=10\;\mathrm{kW}/K$, and τ=5. From Figure 3a, as $\eta_{c}$ raises, ${\bar{E}}_{P_{\max,2}}$, $u_{opt}$, $\pi_{opt}$, ${\bar{P}}_{{\bar{E}}_{P_{\max,2}}}$ and $\eta_{{\bar{E}}_{P_{\max,2}}}$ all raise. When $\eta_{c}$ raises from 0.85 to 1, ${\bar{E}}_{P_{\max,2}}$ raises from 0.4091 to 0.5656, a raise of 38.25%; $u_{opt}$ raises from 0.4679 to 0.4787, a raise of 2.31%; $\pi_{opt}$ raises from 15.3193 to 22.1212, a raise of 44.4%; $P_{{\bar{E}}_{P_{\max,2}}}$ raises from 1.0251 to 1.1982, a raise of 16.89%; $\eta_{{\bar{E}}_{P_{\max,2}}}$ raises from 0.3991 to 0.4720, a raise of 18.27%. This reflects that the value of $\eta_{c}$ has a greater impact on the values of ${\bar{E}}_{P_{\max,2}}$, $\pi_{opt}$, ${\bar{P}}_{{\bar{E}}_{P_{\max,2}}}$ and $\eta_{{\bar{E}}_{P_{\max,2}}}$ and has less impact on the value of $u_{opt}$.

Figure 3b reflects the ${\bar{E}}_{P_{\max,2}}$, the corresponding $u_{opt}$, $\pi_{opt}$, ${\bar{P}}_{{\bar{E}}_{P_{\max,2}}}$ and $\eta_{{\bar{E}}_{P_{\max,2}}}$ vs. $\eta_{t}$ when $\eta_{c}=0.9$, $U_{T}=10\;\mathrm{kW}/K$, and τ=5. From Figure 3b, as $\eta_{t}$ raises, ${\bar{E}}_{P_{\max,2}}$, $u_{opt}$, $\pi_{opt}$, ${\bar{P}}_{{\bar{E}}_{P_{\max,2}}}$ and $\eta_{{\bar{E}}_{P_{\max,2}}}$ raise. When $\eta_{t}$ raises from 0.85 to 1, ${\bar{E}}_{P_{\max,2}}$ raises from 0.3538 to 0.7495, a raise of 111.84%; $u_{opt}$ raises from 0.4651 to 0.4895, a raise of 5.25%; $\pi_{opt}$ raises from 14.7132 to 25.0472, a raise of 70.24%; $P_{{\bar{E}}_{P_{\max,2}}}$ raises from 0.9722 to 1.3098, a raise of 34.73%; $\eta_{{\bar{E}}_{P_{\max,2}}}$ raises from 0.3639 to 0.5722, a raise of 57.24%. This reflects that the value of $\eta_{t}$ has a very large impact on the value of ${\bar{E}}_{P_{\max,2}}$, has a greater impact on the values of $\pi_{opt}$, ${\bar{P}}_{{\bar{E}}_{P_{\max,2}}}$, and $\eta_{{\bar{E}}_{P_{\max,2}}}$, and has the least impact on the value of $u_{opt}$. When $\eta_{c}$ and $\eta_{t}$ gradually raise to 1, the irreversible loss is smaller, and the result is closer to the endoreversible cycle [64], so the ${\bar{E}}_{P_{\max,2}}$ and the $\pi_{opt}$ also raise, and the $u_{opt}$ is closer to 0.5. Comparing the effects of $\eta_{c}$ and $\eta_{t}$ on the ${\bar{E}}_{P}$ performance, it can be found that $\eta_{t}$ has a greater impact on it. Therefore, in the actual project, $\eta_{t}$ improvement should be given priority.

Figure 3c reflects the ${\bar{E}}_{P_{\max,2}}$, the corresponding $u_{opt}$, $\pi_{opt}$, ${\bar{P}}_{{\bar{E}}_{P_{\max,2}}}$ and $\eta_{{\bar{E}}_{P_{\max,2}}}$ vs. $U_{T}$ when $\eta_{c}=\eta_{t}=0.9$, τ=5. From Figure 3c, ${\bar{E}}_{P_{\max,2}}$, as $U_{T}$ raises, ${\bar{E}}_{P_{\max,2}}$, $u_{opt}$, $\pi_{opt}$, ${\bar{P}}_{{\bar{E}}_{P_{\max,2}}}$ and $\eta_{{\bar{E}}_{P_{\max,2}}}$ raise. When $U_{T}$ raises from 2 kW/K to 15 kW/K, ${\bar{E}}_{P_{\max,2}}$ raises from 0.0825 to 0.4720, a raise of 472.12%; $u_{opt}$ raises from 0.3925 to 0.4810, a raise of 22.55%; $\pi_{opt}$ raises from 8.3525 to 17.5940, a raise of 110.64%; $P_{{\bar{E}}_{P_{\max,2}}}$ raises from 0.3221 to 1.1049, a raise of 243.03%; $\eta_{{\bar{E}}_{P_{\max,2}}}$ raises from 0.2561 to 0.4272, a raise of 66.81%. This reflects that the value of $U_{T}$ has a very large impact on the values of ${\bar{E}}_{P_{\max,2}}$ and ${\bar{P}}_{{\bar{E}}_{P_{\max,2}}}$, and has a greater impact on the values of $\pi_{opt}$, $u_{opt}$, and $\eta_{{\bar{E}}_{P_{\max,2}}}$.

Figure 3d reflects the ${\bar{E}}_{P_{\max,2}}$, the corresponding $u_{opt}$, $\pi_{opt}$, ${\bar{P}}_{{\bar{E}}_{P_{\max,2}}}$ and $\eta_{{\bar{E}}_{P_{\max,2}}}$ vs. τ when $\eta_{c}=\eta_{t}=0.9$, $U_{T}=10\;\mathrm{kW}/K$. From Figure 3c, as τ raises, ${\bar{E}}_{P_{\max,2}}$, $\pi_{opt}$, ${\bar{P}}_{{\bar{E}}_{P_{\max,2}}}$ and $\eta_{{\bar{E}}_{P_{\max,2}}}$ raise, $u_{opt}$ reduces. When τ raises from 3 to 5, ${\bar{E}}_{P_{\max,2}}$ raises from 0.0888 to 0.4615, a raise of 419.71%; $\pi_{opt}$ raises from 6.0489 to 17.4299, a raise of 188.15%; $P_{{\bar{E}}_{P_{\max,2}}}$ raises from 0.3310 to 1.0861, a raise of 228.13%; $\eta_{{\bar{E}}_{P_{\max,2}}}$ raises from 0.2683 to 0.4250, a raise of 58.4%; $u_{opt}$ reduces from 0.4752 to 0.4716, a decrease of 0.76%. This reflects that the value of τ has a very large impact on the values of ${\bar{E}}_{P_{\max,2}}$, $\pi_{opt}$ and ${\bar{P}}_{{\bar{E}}_{P_{\max,2}}}$, has a greater impact on the value of $\eta_{{\bar{E}}_{P_{\max,2}}}$, and has the least impact on the value of $u_{opt}$. Increasing the value of τ can greatly improve the performance of cycle ${\bar{E}}_{P}$.

## 4. Multi-Objective Optimizations

**Figure 4:**
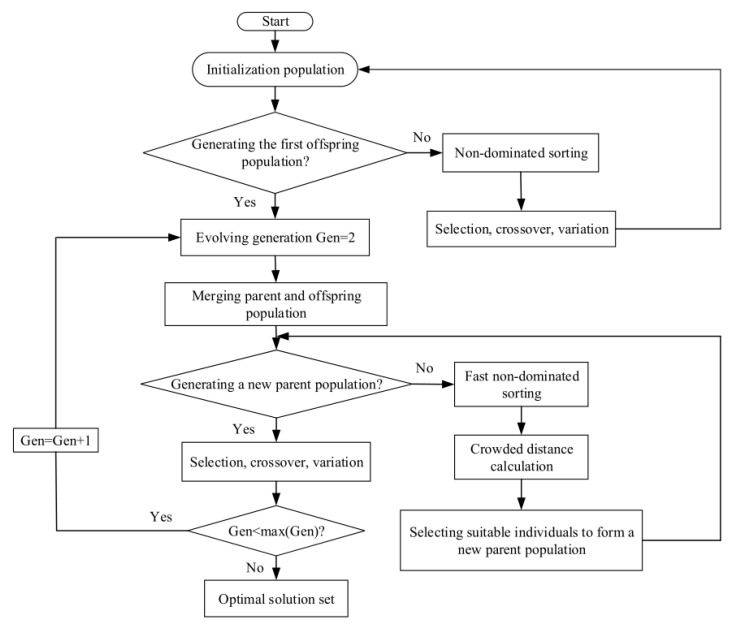
Flow chart of genetic algorithm.

Equations (12) and (16)–(19) are the five performance indicators of irreversible CGT cycle. In actual design, optimization can be carried out according to different requirements, that is, single-objective optimization can be carried out and different objective functions can be combined separately to carry out MOO. In this paper, the NSGA-II is used to implement MOO of the cycle, and three decision methods of Shannon Entropy, TOPSIS and LINMAP are used to select the results with the smallest deviation index (D). Figure 4 is an algorithm flowchart of NSGA-II.

Table 1 lists the comparison of the optimal solutions obtained by MOOs and single-objective optimizations. From Table 1, for five-objective optimization, the Ds obtained by TOPSIS, LINMAP and Shannon Entropy are 0.2921, 0.2921 and 0.2284, respectively. At the maximum $\bar{P}$, η, $\bar{E}$, ${\bar{P}}_{d}$ and ${\bar{E}}_{P}$ conditions, the Ds of five single-objective optimizations are 0.6021, 0.48410, 3836, 0.2427 and 0.2284, respectively.

**Figure 5:**
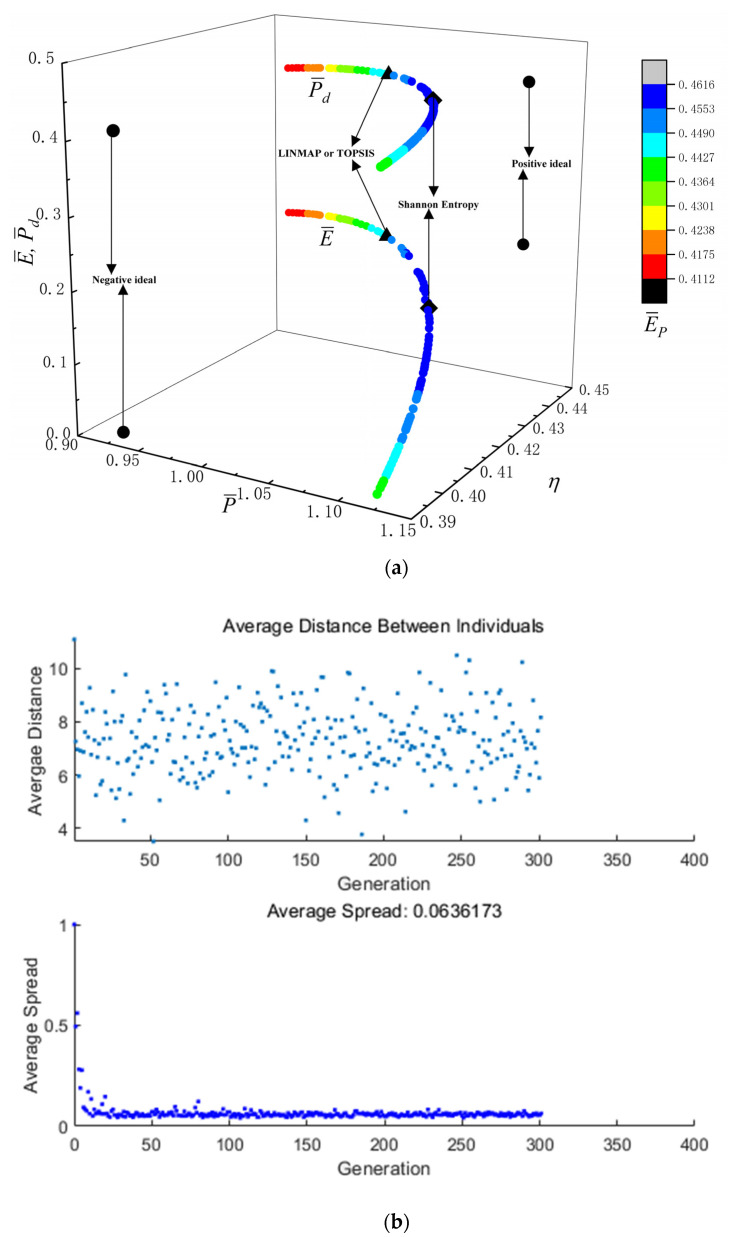

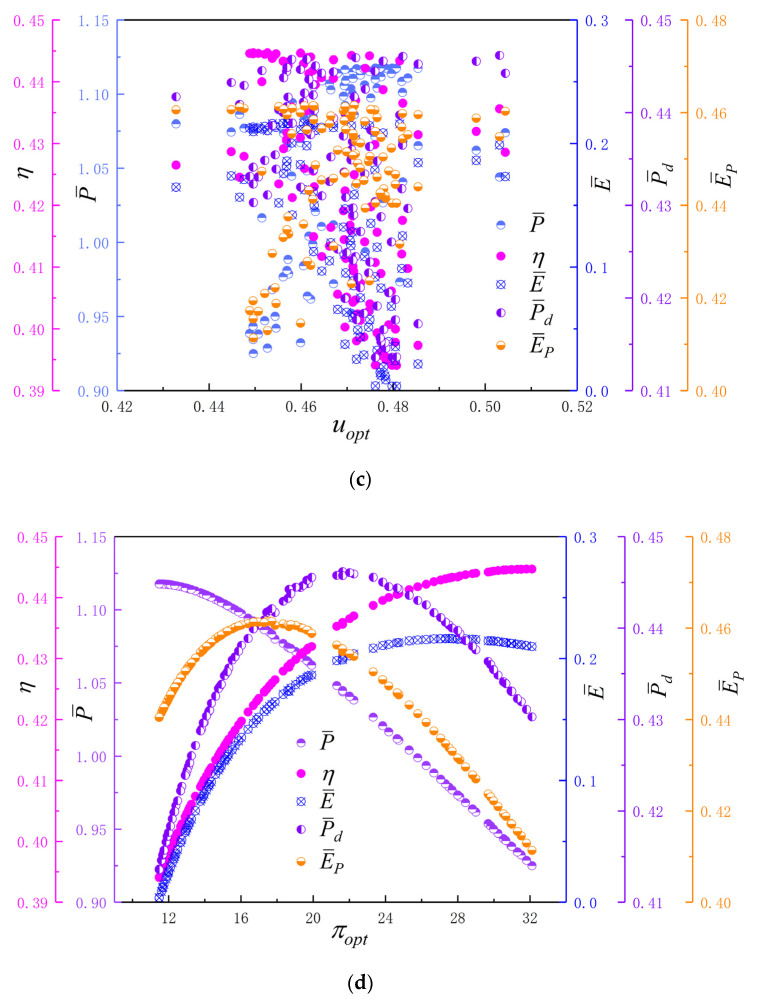
Results of five-objective optimizations. (**a**) $\bar{P}-\eta -\bar{E}-{\bar{P}}_{d}-{\bar{E}}_{P}$; (**b**) Average spread and generation number of $\bar{P}-\eta -\bar{E}-{\bar{P}}_{d}-{\bar{E}}_{P}$; (**c**) $u_{opt}$; (**d**) $\pi_{opt}$.

Figure 5a reflects the Pareto frontier gained by five-objective optimization ($\bar{P}-\eta -\bar{E}-{\bar{P}}_{d}-{\bar{E}}_{P}$). From Figure 5a, as $\bar{P}$ raises, ${\bar{P}}_{d}$ and ${\bar{E}}_{P}$ first raise and then reduce, nd η and $\bar{E}$ reduce. Figure 5b is the average distance generation and average spread generation and converges in the 302th generation when $\bar{P}$, η, $\bar{E}$, ${\bar{P}}_{d}$ and ${\bar{E}}_{P}$ are applied as the OOs for five-objective optimization, and the D acquired by Shannon Entropy approach is 0.2284, which is smaller than the other results. This scheme is more ideal.

Figure 5c,d reflect the distributions of $u_{opt}$ and $\pi_{opt}$ corresponding to the Pareto frontier during optimizations. From Figure 5c, $u_{opt}$ is mainly distributed between 0.45 and 0.49; as $u_{opt}$ raises, the change trends of ${\bar{P}}_{d}$, $\bar{P}$, ${\bar{E}}_{P}$, η and $\bar{E}$ are irregular. From Figure 5d, $\pi_{opt}$ is mainly distributed between 11 and 32; as $\pi_{opt}$ raises, ${\bar{P}}_{d}$ and ${\bar{E}}_{P}$ first raise and then reduce, $\bar{E}$ and η raise, as well as $\bar{P}$ reduces. All of $\pi_{opt}$s corresponding to the maximum ${\bar{P}}_{d}$ and ${\bar{E}}_{P}$ are between 15 and 24.

**Figure 6:**
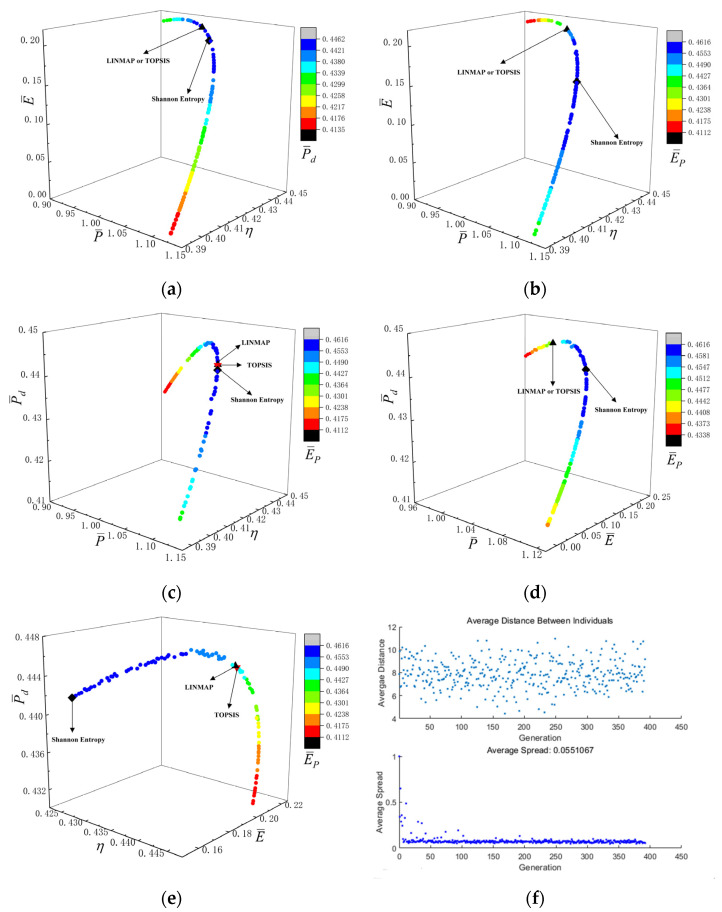
Results of four-objective optimizations. (**a**) $\bar{P}-\eta -\bar{E}-{\bar{P}}_{d}$; (**b**) $\bar{P}-\eta -\bar{E}-{\bar{E}}_{P}$; (**c**) $\bar{P}-\eta -{\bar{P}}_{d}-{\bar{E}}_{P}$; (**d**) $\bar{P}-\bar{E}-{\bar{P}}_{d}-{\bar{E}}_{P}$; (**e**) $\eta -\bar{E}-{\bar{P}}_{d}-{\bar{E}}_{P}$; (**f**) Average spread and generation number of $\bar{P}-\eta -{\bar{P}}_{d}-{\bar{E}}_{P}$.

Figure 6a–e reflect the Pareto frontiers under different four-objective combination optimizations. From Figure 6a–d, as $\bar{P}$ raises, $\bar{E}$, ${\bar{P}}_{d}$ and ${\bar{E}}_{P}$ first raise and then reduce, as well as η reduces. From Figure 6e, as η raises, $\bar{E}$ and ${\bar{P}}_{d}$ first raise and then reduce, as well as ${\bar{E}}_{P}$ reduces. Figure 6f reflects is the average distance generation and average spread generation and converges in the 393th generation when $\bar{P}$, η, ${\bar{P}}_{d}$ and ${\bar{E}}_{P}$ are applied as the OOs for four-objective optimization, and the D acquired by LINMAP approach is 0.2163, which is smaller than the other results. This scheme is more ideal.

**Figure 7:**
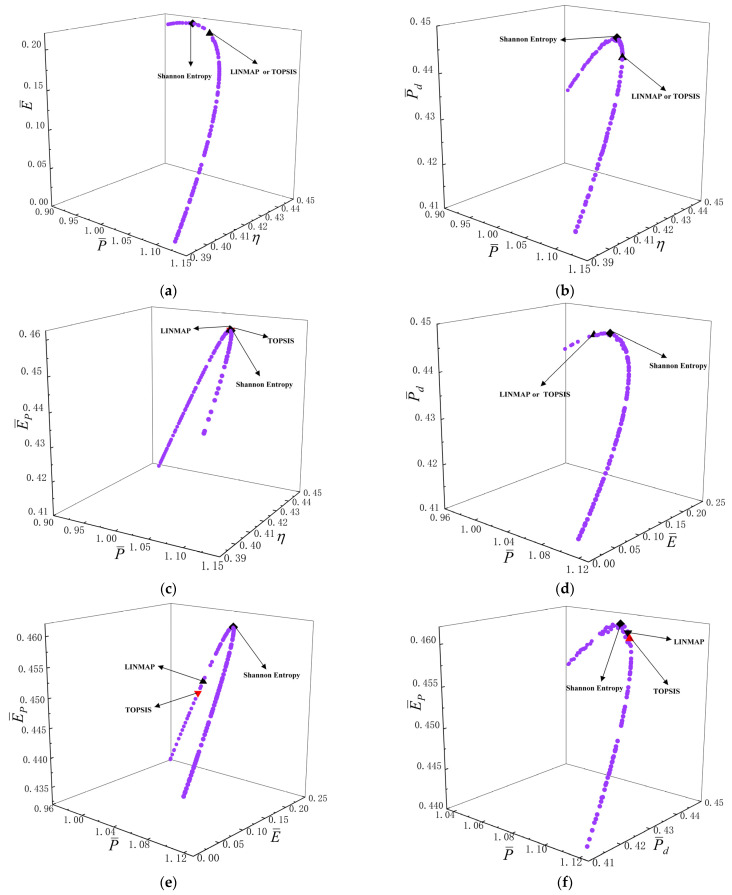

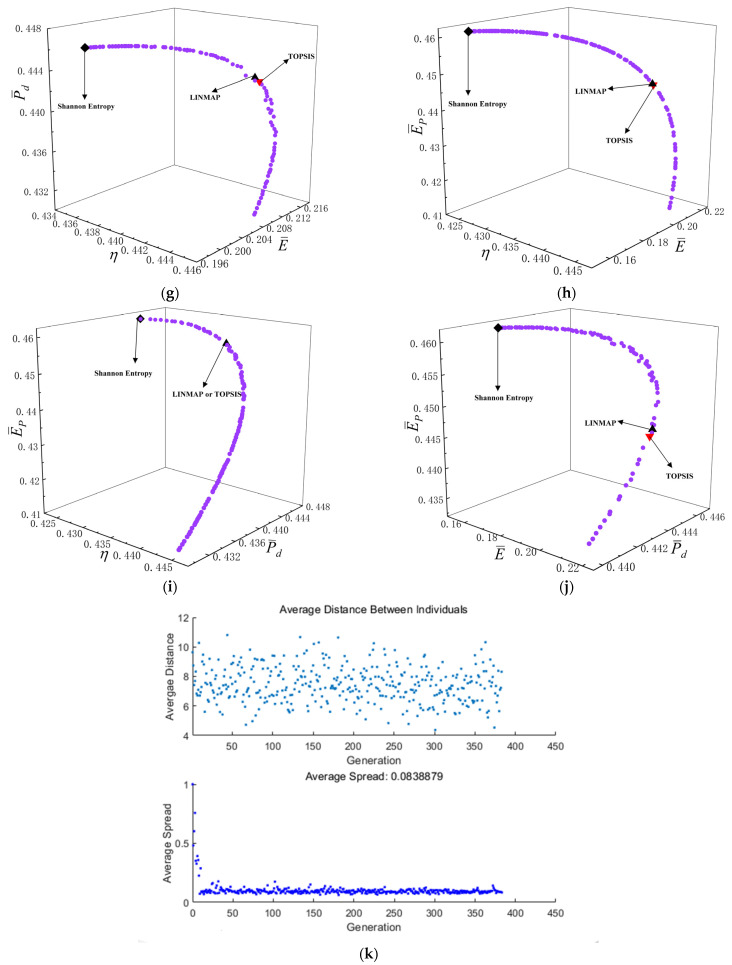
Results of three-objective optimizations. (**a**) $\bar{P}-\eta -\bar{E}$; (**b**) $\bar{P}-\eta -{\bar{P}}_{d}$; (**c**) $\bar{P}-\eta -{\bar{E}}_{P}$; (**d**) $\bar{P}-\bar{E}-{\bar{P}}_{d}$; (**e**) $\bar{P}-\bar{E}-{\bar{E}}_{P}$; (**f**) $\bar{P}-{\bar{P}}_{d}-{\bar{E}}_{P}$; (**g**) $\eta -\bar{E}-{\bar{P}}_{d}$; (**h**) $\eta -\bar{E}-{\bar{E}}_{P}$; (**i**) $\eta -{\bar{P}}_{d}-{\bar{E}}_{P}$; (**j**) $\bar{E}-{\bar{P}}_{d}-{\bar{E}}_{P}$; (**k**) Average spread and generation number of $\bar{P}-\eta -{\bar{E}}_{P}$.

Figure 7a–j reflect the Pareto frontiers under different three-objective combination optimizations. From Figure 7a–f, as $\bar{P}$ raises, $\bar{E}$, ${\bar{P}}_{d}$ and ${\bar{E}}_{P}$ first raise and then reduce, and η reduces. From Figure 7g–i, as η raises, $\bar{E}$ first raises and then reduces, as well as ${\bar{E}}_{P}$ and ${\bar{P}}_{d}$ reduce. From Figure 7j, as $\bar{E}$ raises, ${\bar{P}}_{d}$ first raises and then reduces, as well as ${\bar{E}}_{P}$ reduces. Figure 7k is the average distance generation and average spread generation and converges in the 384th generation when $\bar{P}$, η and ${\bar{E}}_{P}$ are applied as the OOs for three-objective optimization and the D acquired by LINMAP approach is 0.2067, which is smaller than the other results. This scheme is more ideal.

**Figure 8:**
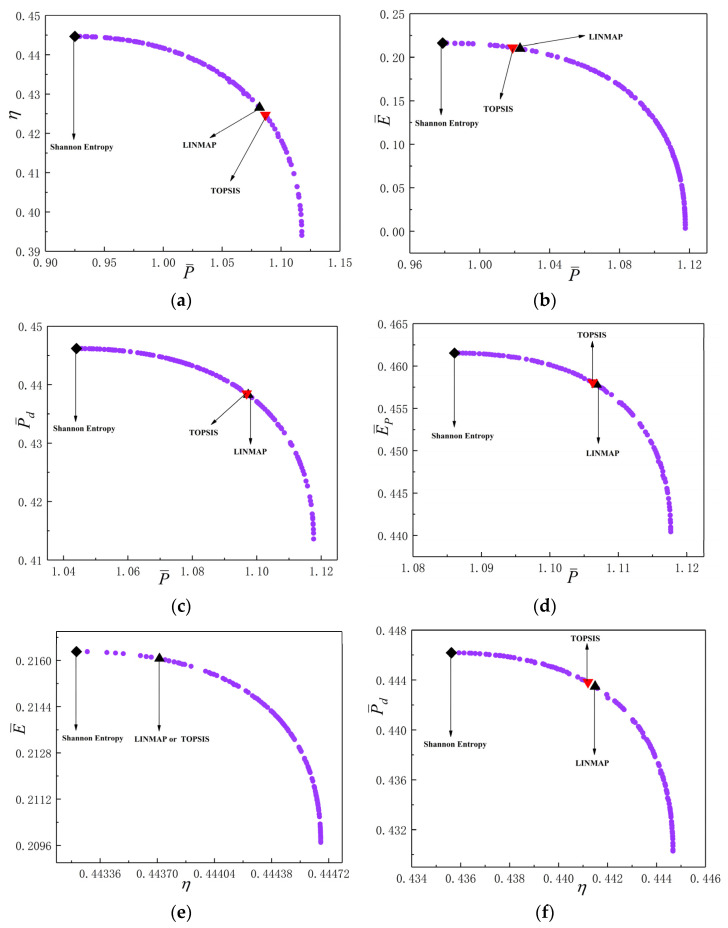

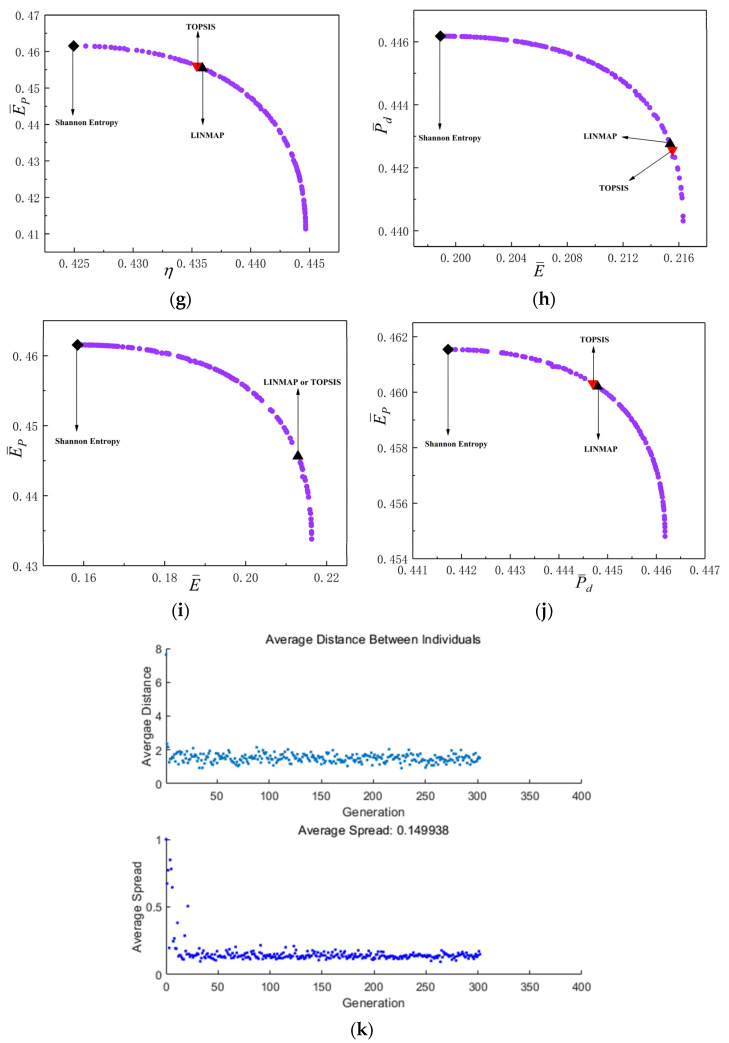
Results of two-objective optimizations. (**a**) $\bar{P}-\eta$; (**b**) $\bar{P}-\bar{E}$; (**c**) $\bar{P}-{\bar{P}}_{d}$; (**d**) $\bar{P}-{\bar{E}}_{P}$; (**e**) $\eta -\bar{E}$; (**f**) $\eta -{\bar{P}}_{d}$; (**g**) $\eta -{\bar{E}}_{P}$; (**h**) $\bar{E}-{\bar{P}}_{d}$; (**i**) $\bar{E}-{\bar{E}}_{P}$; (**j**) ${\bar{P}}_{d}-{\bar{E}}_{P}$; (**k**) Average spread and generation number of ${\bar{P}}_{d}-{\bar{E}}_{P}$.

Figure 8a–j reflect the Pareto frontiers under different two-objective combinations. From Figure 8a–d, as $\bar{P}$ raises, η, $\bar{E}$, ${\bar{P}}_{d}$ and ${\bar{E}}_{P}$ all reduce. From Figure 8e–g, as η raises, $\bar{E}$, ${\bar{P}}_{d}$ and ${\bar{E}}_{P}$ reduce. From Figure 8h,i, as $\bar{E}$ raises, ${\bar{P}}_{d}$ and ${\bar{E}}_{P}$ reduce. From Figure 8j, as ${\bar{P}}_{d}$ raises, ${\bar{E}}_{P}$ reduces. Figure 8k is the average distance generation and average spread generation and converges in the 303th generation when ${\bar{P}}_{d}$ and ${\bar{E}}_{P}$ are applied as the OOs for two-objective optimization, and the D acquired by LINMAP approach is 0.2060, which is smaller than the other results. This scheme is more ideal.

## 5. Conclusions

Based on the simple irreversible CGT cycle model established in Refs. [54,60], this paper derives the ${\bar{E}}_{P}$ expression of the irreversible CGT cycle. When the $U_{T}$ is constant, ${\bar{E}}_{P_{\max,2}}$ is obtained by optimizing u and π. Applying NSGA-II to carry out MOO on five OOs of $\bar{P}$, η, $\bar{E}$, ${\bar{P}}_{d}$ and ${\bar{E}}_{P}$ and using TOPSIS, LINMAP and Shannon Entropy strategies to gain deviation indexes of MOO on different combinations of OOs, the results reflect that:

1. When the $U_{T}$ is constant, the existence of both $u_{opt}$ and $\pi_{opt}$ make cycle ${\bar{E}}_{P}$ reach a quadratic maximum (${\bar{E}}_{P_{\max,2}}$); with the raises of $\eta_{c}$, $\eta_{t}$, $U_{T}$ and τ, cycle ${\bar{E}}_{P}$ has a significant raise, of which $\eta_{t}$ and τ have great impacts on ${\bar{E}}_{P}$.
2. For five-objective optimization, the D obtained by the Shannon Entropy decision-making method is 0.2284, which is better than other decision-making methods.
3. For four-objective combination optimizations, the D obtained by LINMAP decision-making method with four-objective optimization of $\bar{P}$, η, ${\bar{P}}_{d}$ and ${\bar{E}}_{P}$ is 0.2163, which is better than other four-objective combination optimizations.
4. For three-objective combination optimizations, the D obtained by LINMAP decision-making method with three-objective optimization of $\bar{P}$, η and ${\bar{E}}_{P}$ is 0.2067, which is better than other three-objective combination optimizations.
5. For two-objective combination optimization, the D obtained by LINMAP decision-making method with two-objective optimization of ${\bar{P}}_{d}$ and ${\bar{E}}_{P}$ is 0.2060, which is better than other two-objective combination optimizations.
6. FTT and NSGA-II are powerful theoretical and computational tools for comprehensive performance optimization of a simple irreversible CGT cycle.

## Figures and Tables

**Table 1:** Comparisons of five-, four-, three-, two- and single-objective optimization results.

OOs	Decision-Making Methods	Optimization Variables		Performance Indicators					Deviation Index
		u	π	$\bar{P}$	η	$\bar{E}$	${\bar{P}}_{d}$	${\bar{E}}_{P}$	D
$\bar{P}$, η, $\bar{E}$, ${\bar{P}}_{d}$ and ${\bar{E}}_{P}$	LINMAP	0.4631	23.8439	1.0208	0.4395	0.2106	0.4450	0.4487	0.2921
	TOPSIS	0.4631	23.8439	1.0208	0.4395	0.2106	0.4450	0.4487	0.2921
	Shannon Entropy	0.4716	17.4295	1.0861	0.4250	0.1585	0.4417	0.4615	0.2284
$\bar{P}$, η, $\bar{E}$ and ${\bar{P}}_{d}$	LINMAP	0.4705	24.1718	1.0172	0.4399	0.2115	0.4449	0.4475	0.3003
	TOPSIS	0.4705	24.1718	1.0172	0.4399	0.2115	0.4449	0.4475	0.3003
	Shannon Entropy	0.5031	21.6376	1.0440	0.4356	0.1989	0.4462	0.4548	0.2428
$\bar{P}$, η, $\bar{E}$ and ${\bar{E}}_{P}$	LINMAP	0.4630	23.5611	1.0239	0.4391	0.2096	0.4452	0.4496	0.2850
	TOPSIS	0.4630	23.5611	1.0239	0.4391	0.2096	0.4452	0.4496	0.2850
	Shannon Entropy	0.4716	17.4295	1.0861	0.4250	0.1585	0.4417	0.4615	0.2284
$\bar{P}$, η, ${\bar{P}}_{d}$ and ${\bar{E}}_{P}$	LINMAP	0.4664	17.9547	1.0814	0.4267	0.1658	0.4427	0.4617	0.2163
	TOPSIS	0.4785	17.7990	1.0829	0.4261	0.1635	0.4426	0.4615	0.2198
	Shannon Entropy	0.4716	17.4295	1.0861	0.4250	0.1585	0.4417	0.4615	0.2284
$\bar{P}$, $\bar{E}$, ${\bar{P}}_{d}$ and ${\bar{E}}_{P}$	LINMAP	0.4617	23.9764	1.0193	0.4397	0.2110	0.4449	0.4482	0.2955
	TOPSIS	0.4617	23.9764	1.0193	0.4397	0.2110	0.4449	0.4482	0.2955
	Shannon Entropy	0.4716	17.4295	1.0861	0.4250	0.1585	0.4417	0.4615	0.2284
η, $\bar{E}$, ${\bar{P}}_{d}$ and ${\bar{E}}_{P}$	LINMAP	0.4584	24.4045	1.0144	0.4403	0.2123	0.4445	0.4466	0.3066
	TOPSIS	0.4559	24.5374	1.0128	0.4404	0.2127	0.4443	0.4461	0.3102
	Shannon Entropy	0.4716	17.4295	1.0861	0.4250	0.1585	0.4417	0.4615	0.2284
$\bar{P}$, η and $\bar{E}$	LINMAP	0.4662	23.5299	1.0243	0.4399	0.2094	0.4453	0.4498	0.2839
	TOPSIS	0.4662	23.5299	1.0243	0.4399	0.2094	0.4453	0.4498	0.2839
	Shannon Entropy	0.4572	27.5112	0.9789	0.4432	0.2163	0.4403	0.4339	0.3836
$\bar{P}$, η and ${\bar{P}}_{d}$	LINMAP	0.4604	18.3981	1.0771	0.4281	0.1715	0.4433	0.4611	0.2099
	TOPSIS	0.4604	18.3981	1.0771	0.4281	0.1715	0.4433	0.4611	0.2099
	Shannon Entropy	0.5033	21.6266	1.0441	0.4356	0.1988	0.4462	0.4548	0.2427
$\bar{P}$, η and ${\bar{E}}_{P}$	LINMAP	0.4688	18.1390	1.0797	0.4373	0.1682	0.4431	0.4613	0.2067
	TOPSIS	0.4693	17.6281	1.0843	0.4256	0.1614	0.4421	0.4615	0.2233
	Shannon Entropy	0.4716	17.4295	1.0861	0.4250	0.1585	0.4417	0.4615	0.2284
$\bar{P}$, $\bar{E}$ and ${\bar{P}}_{d}$	LINMAP	0.4688	18.1390	1.0797	0.4273	0.1682	0.4431	0.4613	0.2131
	TOPSIS	0.4693	17.6281	1.0843	0.4256	0.1614	0.4421	0.4615	0.2233
	Shannon Entropy	0.4714	17.4303	1.0861	0.4250	0.1585	0.4417	0.4615	0.2284
$\bar{P}$, $\bar{E}$ and ${\bar{E}}_{P}$	LINMAP	0.4661	23.4256	1.0255	0.4389	0.2091	0.4453	0.4501	0.2814
	TOPSIS	0.4671	24.1141	1.0178	0.4399	0.2114	04449	0.4477	0.5000
	Shannon Entropy	0.4716	17.4295	1.0861	0.4250	0.1585	0.4417	0.4615	0.2284
$\bar{P}$, ${\bar{P}}_{d}$ and ${\bar{E}}_{P}$	LINMAP	0.4923	15.9844	1.0976	0.4194	0.1339	0.4382	0.4604	0.2836
	TOPSIS	0.4892	16.3692	1.0947	0.4210	0.1410	0.4393	0.4609	0.2666
	Shannon Entropy	0.4716	17.4313	1.0861	0.4250	0.1585	0.4417	0.4615	0.2284
η, $\bar{E}$ and ${\bar{P}}_{d}$	LINMAP	0.4743	26.2934	0.9932	0.4422	0.2155	0.4425	0.4392	0.3536
	TOPSIS	0.4689	26.5421	0.9903	0.4425	0.2158	0.4421	0.4382	0.3597
	Shannon Entropy	0.5033	21.6266	1.0441	0.4356	0.1988	0.4462	0.4548	0.2427
η, $\bar{E}$ and ${\bar{E}}_{P}$	LINMAP	0.4712	24.7000	1.0113	0.4406	0.2129	0.4444	0.4456	0.3137
	TOPSIS	0.4684	24.7747	1.0104	0.4407	0.2132	0.4443	0.4453	0.3156
	Shannon Entropy	0.4716	17.4295	1.0861	0.4250	0.1585	0.4417	0.4615	0.2284
η, ${\bar{P}}_{d}$ and ${\bar{E}}_{P}$	LINMAP	0.4667	21.6485	1.0448	0.4359	0.2003	0.4459	0.4554	0.2388
	TOPSIS	0.4667	21.6485	1.0448	0.4359	0.2003	0.4459	0.4554	0.2388
	Shannon Entropy	0.4716	17.4295	1.0861	0.4250	0.1585	0.4417	0.4615	0.2284
$\bar{E}$, ${\bar{P}}_{d}$ and ${\bar{E}}_{P}$	LINMAP	0.4662	24.4018	1.0146	0.4002	0.2123	0.4446	0.4467	0.3281
	TOPSIS	0.4625	24.6222	1.0120	0.4005	0.2129	0.4443	0.4458	0.3333
	Shannon Entropy	0.4716	17.4295	1.0861	0.4250	0.1585	0.4417	0.4615	0.2284
$\bar{P}$ and η	LINMAP	0.4728	17.9202	1.0818	0.4266	0.1653	0.4427	0.4614	0.2169
	TOPSIS	0.4806	17.3594	1.0867	0.4247	0.1572	0.4417	0.4615	0.2311
	Shannon Entropy	0.4496	32.1083	0.9250	0.4447	0.2097	0.4303	0.4113	0.4841
$\bar{P}$ and $\bar{E}$	LINMAP	0.4613	23.6354	1.0230	0.4392	0.2099	0.4451	0.4494	0.2871
	TOPSIS	0.4724	24.0275	1.0188	0.4397	0.2111	0.4450	0.4480	0.2967
	Shannon Entropy	0.4572	27.5112	0.9789	0.4432	0.2163	0.4403	0.4339	0.3836
$\bar{P}$ and ${\bar{P}}_{d}$	LINMAP	0.4890	16.0275	1.0974	0.4196	0.1348	0.4383	0.4605	0.2812
	TOPSIS	0.4925	16.0781	1.0969	0.4198	0.1356	0.4385	0.4605	0.2795
	Shannon Entropy	0.5031	21.6370	1.0441	0.4356	0.1988	0.4462	0.4548	0.2427
$\bar{P}$ and ${\bar{E}}_{P}$	LINMAP	0.4758	14.6596	1.1068	0.4136	0.1062	0.4330	0.4578	0.4741
	TOPSIS	0.4738	14.7508	1.1050	0.4150	0.1131	0.4342	0.4586	0.4653
	Shannon Entropy	0.4720	17.4284	1.0861	0.4250	0.1585	0.4417	0.4615	0.2284
η and $\bar{E}$	LINMAP	0.4552	28.3443	0.9692	0.4437	0.2161	0.4388	0.4300	0.4033
	TOPSIS	0.4554	28.2543	0.9702	0.4437	0.2161	0.4390	0.4350	0.3994
	Shannon Entropy	0.4571	27.5193	0.9789	0.4432	0.2163	0.4403	0.4339	0.3836
η and ${\bar{P}}_{d}$	LINMAP	0.4732	25.5262	1.0019	0.4415	0.2146	0.4435	0.4423	0.3346
	TOPSIS	0.4837	25.3135	1.0042	0.4412	0.2139	0.4438	0.4431	0.3299
	Shannon Entropy	0.5031	21.6370	1.0441	0.4356	0.1988	0.4462	0.4548	0.2427
η and ${\bar{E}}_{P}$	LINMAP	0.4735	21.6481	1.0448	0.4359	0.2002	0.4460	0.4554	0.2389
	TOPSIS	0.4614	21.3699	1.0476	0.4354	0.1986	0.4458	0.4561	0.2331
	Shannon Entropy	0.4720	17.4284	1.0861	0.4250	0.1585	0.4417	0.4615	0.2284
$\bar{E}$ and ${\bar{P}}_{d}$	LINMAP	0.4693	26.0511	0.9959	0.4420	0.2154	0.4428	0.4402	0.3477
	TOPSIS	0.4674	26.1729	0.9945	0.4421	0.2155	0.4426	0.4397	0.3507
	Shannon Entropy	0.5031	21.6347	1.0441	0.4356	0.1988	0.4462	0.4548	0.2427
$\bar{E}$ and ${\bar{E}}_{P}$	LINMAP	0.4705	24.6813	1.0115	0.4406	0.2129	0.4444	0.4456	0.3132
	TOPSIS	0.4705	24.6813	1.0115	0.4406	0.2129	0.4444	0.4456	0.3132
	Shannon Entropy	0.4716	17.4301	1.0861	0.4250	0.1585	0.4417	0.4615	0.2284
${\bar{P}}_{d}$ and ${\bar{E}}_{P}$	LINMAP	0.4814	19.2494	1.0693	0.4304	0.1806	0.4448	0.4602	0.2060
	TOPSIS	0.4849	19.1263	1.0704	0.4300	0.1792	0.4447	0.4603	0.2064
	Shannon Entropy	0.4716	17.4295	1.0861	0.4250	0.1585	0.4417	0.4615	0.2284
$\bar{P}$		0.4798	11.4757	1.1177	0.3941	0.0036	0.4136	0.4404	0.6021
η		0.4496	32.1061	0.9250	0.4447	0.2097	0.4303	0.4113	0.4841
$\bar{E}$		0.4573	27.5224	0.9789	0.4432	0.2163	0.4403	0.4339	0.3836
${\bar{P}}_{d}$		0.5031	21.6324	1.0441	0.4356	0.1988	0.4462	0.4548	0.2427
${\bar{E}}_{P}$		0.4716	17.4295	1.0861	0.4250	0.1585	0.4417	0.4615	0.2284
Positive ideal point Negative ideal point				1.1177	0.4447	0.2163	0.4462	0.4615	
				0.9251	0.3941	0.0035	0.4136	0.4113	

## Data Availability

Not applicable.

## Nomenclature

C	Thermal capacity ratio (kW/K)
D	Deviation index
E	Effectiveness of heat exchanger or ecological function (kW)
$E_{P}$	Efficient power (kW)
k	Specific heat ratio
N	Number of the heat transfer unit
P	Power output (kW)
$P_{d}$	Power density (${\mathrm{kW}/m}^{3}$)
p	Pressure (kPa)
Q	Heat absorbing rate or heat releasing rate (kW)
$S_{g}$	Entropy production rate (kW/K)
T	Temperature (K)
$T_{0}$	Ambient temperature (K)
U	Heat conductance (kW/K)
$U_{T}$	Total heat conductance (kW/K)
u	Distribution of hot-side heat exchanger heat conductance
v	Volume ($m^{3}$)
Greek symbols	
η	Thermal efficiency
π	Pressure ratio
τ	Heat reservoirs temperature ratio
Subscripts	
c	Compressor
${\bar{E}}_{P_{\max,2}}$	Double maximum dimensionless efficient power point
H	Hot-side
L	Cold-side
max	Maximum value
max,2	Double maximum value
opt	Optimal
$\bar{P}$	Maximum dimensionless power point
t	Turbine
wf	Working fluid
1–4, $2_{s}$, $4_{s}$	Cycle state points
Superscripts	
−	Dimensionless
**Abbreviations**	
CGT	Closed gas turbine
FTT	Finite-time thermodynamic
HEG	Heat engine
HEX	Heat exchanger
HTC	Heat conductance
MOO	Multi-objective optimization
OO	Optimization objective
